# CMOS Image Sensors for High Speed Applications

**DOI:** 10.3390/s90100430

**Published:** 2009-01-13

**Authors:** Munir El-Desouki, M. Jamal Deen, Qiyin Fang, Louis Liu, Frances Tse, David Armstrong

**Affiliations:** 1 Department of Electrical and Computer Engineering, McMaster University, Hamilton, ON, Canada; E-Mail: eldesom@mcmaster.ca; 2 Department of Engineering Physics, McMaster University, Hamilton, ON, Canada; E-Mail: qfang@mcmaster.ca; 3 Department of Medicine, University of Toronto, Toronto, ON, Canada; E-Mail: louis.liu@uhn.on.ca; 4 Department of Medicine, McMaster University, Hamilton, ON, Canada; E-Mails: tsef@mcmaster.ca; armstro@mcmaster.ca

**Keywords:** Active-pixel-sensor, biomedical imaging, CCD, CMOS, high-speed, image-sensor, photodetectors, smart-pixel

## Abstract

Recent advances in deep submicron CMOS technologies and improved pixel designs have enabled CMOS-based imagers to surpass charge-coupled devices (CCD) imaging technology for mainstream applications. The parallel outputs that CMOS imagers can offer, in addition to complete camera-on-a-chip solutions due to being fabricated in standard CMOS technologies, result in compelling advantages in speed and system throughput. Since there is a practical limit on the minimum pixel size (4∼5 μm) due to limitations in the optics, CMOS technology scaling can allow for an increased number of transistors to be integrated into the pixel to improve both detection and signal processing. Such smart pixels truly show the potential of CMOS technology for imaging applications allowing CMOS imagers to achieve the image quality and global shuttering performance necessary to meet the demands of ultrahigh-speed applications. In this paper, a review of CMOS-based high-speed imager design is presented and the various implementations that target ultrahigh-speed imaging are described. This work also discusses the design, layout and simulation results of an ultrahigh acquisition rate CMOS active-pixel sensor imager that can take 8 frames at a rate of more than a billion frames per second (fps).

## 1. Introduction

Emerging imaging applications, such as integral machine vision, time-of-flight (TOF) imaging, topographic imaging, three-dimensional high-definition television (3D-HDTV) and optical molecular imaging systems, specifically fluorescence life-time imaging (FLIM), have resulted in significant research efforts in designing high-speed imagers [1-17]. The advances in deep submicron CMOS technologies have especially made such high-speed imaging possible. One of the main advantages of CMOS image sensors is that they are fabricated in standard CMOS technologies, which allows for full integration of the image sensor along with the processing and control circuits on the same chip and at a low cost. This camera-on-chip system leads to reduction in power consumption, cost and sensor size and allows for integration of new sensor functionalities. Since there is a practical limit on the minimum pixel size (4∼5 μm), CMOS technology scaling can allow for an increased number of transistors to be integrated. For example, when using a CMOS 0.18 μm technology with a 5 μm × 5 μm pixel and a 30% fill-factor (FF), eight analog transistors or 32 digital transistors can be integrated within the pixel. Since digital transistors take more advantage of CMOS scaling properties, digital pixel sensors (DPS) have become very attractive. Such smart pixels truly show the potential of CMOS technology for imaging applications allowing CMOS imagers to achieve the image quality and global shuttering performance necessary to meet the demands of ultrahigh-speed applications. Such applications include biometric analysis, robotic visions systems, material analysis, *in-vivo* bio-imaging, human-interfaces and geological surveying.

**Figure 1.**
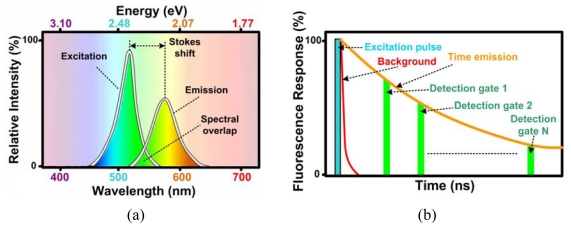
(a) Fluorescence spectral response showing the excitation pulse and the emission pulse. (b) Time-resolved and fluorescence lifetime measurements [2].

A common optical imaging technique used for *in-vivo* bio-imaging and medical characterization is fluorescence imaging. Fluorescence is the property of certain atoms and molecules to absorb light at a particular wavelength and emit light at a longer wavelength [Figure 1 (a)], over a short interval of time known as the fluorescence lifetime. Immediately following excitation, the fluorescence intensity decays exponentially, usually over a few nanoseconds for most biological fluorophores [2, 18].

When testing molecules that have overlapping spectra, such as cancerous and non-cancerous cells, one valuable method is time-resolved measurements such as FLIM. Time resolved techniques are used to determine the relaxation times of fluorescence signals. Since the signal has an exponential decay over time, integrating approaches that have integration times much longer than the average fluorescent lifetime cannot be used. Rather, averaging a number of repeated measurements in narrow sampling windows or gates [Figure 1 (b)] have been shown to be more effective [19]. Such high frame-rate applications require a fast and sensitive CMOS imager. CMOS imagers that can achieve timing resolutions between 150-800 ps from 64×64 pixel imagers with two point per transient waveform sampling and 150 fps, have been reported in the literature [19]. In order to sample a fluorescence lifetime curve without using repeated experiments, a CMOS imager that can capture a number of consecutive frames at sub-nanosecond resolution would be required. The photodiode must be very sensitive as well, which may require the use of avalanche-photodiodes (APD) [20].

Another high-speed imaging application is proton radiography [21], which is a new tool for advanced hydrotesting. Proton radiography had recently become an attractive imaging tool when the blurry images that would result from proton scattering were improved by using magnetic lens to focus the protons. Proton radiography is especially attractive when imaging thick objects and acquiring images at high frame-rates (5 million fps). Image sensors used for proton radiography must be able to capture images at rates of thousands to millions of frames-per-second, even if only for a few frames [21]. The reader is refereed to [1] and [22] for a review of various high-speed imaging application requirements.

**Figure 2.**
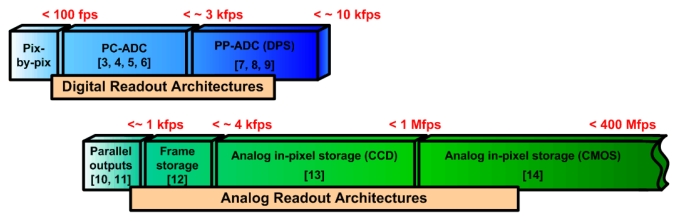
A block-diagram categorizing some of the most relevant published high-speed imagers [3-14], showing the frame rates that can be achieved by different readout architectures.

This paper presents a review of the existing CMOS high-speed imager designs and discusses the various implementations that target ultrahigh-speed imaging. Figure 2 shows a block-diagram categorizing some of the high-speed imagers [3-14], showing the frame rates (FR) that can be achieved with different readout architectures.

The readout architectures that are discussed in this paper include the standard pixel-by-pixel sequential readout, the per-column analog-to-digital converter (PC-ADC) readout and the per-pixel ADC (PP-ADC), in addition to the analog techniques used for high-speed imagers. This work also discusses the design, layout and simulation results of an ultrahigh acquisition rate CMOS active-pixel sensor imager that can take 8 frames at a rate of more than a billion fps. The design is simulated in the IBM 130 nm standard CMOS technology. The blocks in Figure 2 will be explained in the following sections of this paper, which is organized as follows. Section 2 discusses the various digital readout architectures, followed by the analog readout architectures in Section 3. Section 4 discusses the ultrahigh-speed pixel design and simulation results that can take over one billion fps, which is followed by the conclusions in Section 5.

### 2. Digital Readout Architectures

**Figure 3.**
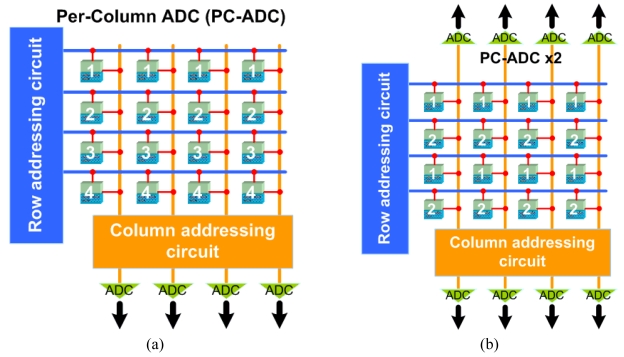
Array access in a simple pixel-by-pixel (PBP) sequential readout architecture.

Over the past few years, a number of readout architectures have been used for CMOS imagers [3-9]. Referring back to Figure 2, the simplest and slowest form of readout is sequential pixel-by-pixel (PBP) array access. Figure 3 shows the sequence of pixel access in such an array.

The frame rate (*FR*) in this case can be calculated as:

(1) $$FR_{\text{PBP}}={\left[H\times V\left(\tau_{\text{PBP}}+\frac{b}{n}\times \tau_{\text{RO}}\right)\right]}^{-1}$$

where *H* and *V* are the number of rows and columns in the array respectively, *τ_ADC_* is the time it takes the ADC to complete one conversion, *τ_RO_* is the time it takes the chip I/O to send out the converted digital result, *b* is the number of digital bits, and *n* is the number of parallel outputs. The dominating factors in equation (1) are the *H*×*V* product and *τ_ADC_*, which shows that this architecture cannot be used for high resolutions. For example, with a *τ_ADC_* of 2 μ s, the FR drops below 30 fps for an imager of 128×128 and drops below 0.5 fps for a 1M-pixel imager. It is worth mentioning, however, that the pixel-by-pixel readout architecture has the lowest fixed-pattern-noise (FPN) of the three readout architectures.

The FR discussion presented in this paper does not include the integration time because it assumes that the integration time is fixed for all pixels and in all three readout architectures, which is the case in voltage-domain imagers. On the other hand, time-domain imagers [23-27] exhibit a tradeoff between integration time and dynamic-range, whereas the tradeoff with dynamic-range in voltage-domain imagers is with the DC supply voltage. Kitchen *et al.* [23] define the dynamic range of a time-domain imager as a function of the ratio between the maximum to minimum integration times. Depending on the dynamic-range required for a specific application, the maximum integration time can be the dominating factor of the FR, making time-domain imagers unsuitable for ultrahigh-speed imaging applications. However, time-domain imagers are a good choice when dealing with application that require very high dynamic-ranges (∼100 dB) [23-27] and low-power consumption since they can operate from lower supply voltages compared to voltage-domain imagers. The following subsections discuss the efforts presented in the literature to achieve high-speed imaging on the array-level as well as the pixel-level.

### 2.1. Array-Level Techniques

**Figure 4.**
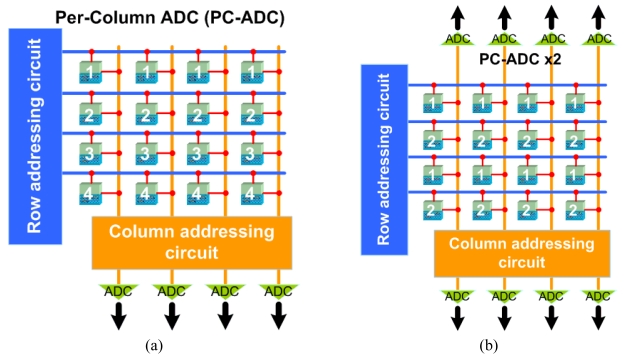
Array access in (a) a per-column ADC (PC-ADC) readout and (b) a PC-ADC ×2.

One of the most common techniques to increase the FR is to process in parallel as many pixels in the array as possible. The most common technique to do so is to have an ADC per every column of the array [3-6], as shown in Figure 4 (a).

The FR of a PC-ADC design is almost *V* times faster than a sequential readout array, which comes at the expense of an increase in power consumption and silicon area. The *FR* is calculated as:

(2) $${\text{FR}}_{\text{PC}-\text{ADC}}={\left[H\times V\left(\frac{\tau_{\text{ADC}}}{V}+\frac{b}{n}\times \tau_{\text{RO}}\right)\right]}^{-1}$$

Krymski *et al.* [3] described a 1M-pixel imager in 1999 using a 0.5 μ m CMOS technology that has a FR of 500 fps. The array of 1,024(*H*)×1,024(*V*) pixels had a PC-ADC architecture that was divided into two groups of ADCs on the top and the bottom of the array since the pixel pitch was very small (10 μ m). The authors used a dual-port RAM to double the readout speed since writing to the RAM from the 8-bit ADCs (*b*=8) and readout can be done simultaneously. The authors also used 8 output ports of 8-bits each in parallel (*n*=64) to send out the data clocked at a master clock rate of 66 MHz (1/*τ_RO_*). The ADC conversion time in addition to the sample time was 2 μ s (*τ_ADC_*). By substituting these numbers into equation (2), a FR of 248 fps can be found, which is doubled due to the dual-port RAM, as reported by the authors [3]. This high FR comes at the expense of a power consumption of 350 mW from a 3.3 V supply. If the imager in [3] was designed using a pixel-by-pixel architecture, from equation (1) the FR can be calculated to be 0.5 fps, which would be doubled due to the dual-port RAM to only 1 fps. In 2003, the same group improved this work using a smaller feature size of 0.35 μ m CMOS technology and increased the imager and ADC resolution to 4.1M-pixels and 10-bits with a FR of 240 fps [4]. With such high resolution, although the FR is not very high, this imager delivers 9.75 Gb/s of data. This shows how the bottleneck for high resolution imagers can be the chip I/O transfer. Nishikawa *et al.* [5] reported an on-chip parallel image compression circuit to address the I/O bottleneck. With the proposed compression technique and a master clock rate of 53 MHz, the authors propose a 3,000 fps 1M-pixel imager [5].

Another way to increase parallelism and improve the FR is to split the array into two groups and have a group of top ADCs and a group of bottom ADCs, each in charge of reading out half of the array, as shown in Figure 4 (b). This technique is more feasible in biomedical arrays where the pixel pitch is large due to needing large photodiodes to increase the sensitivity [19, 28]. A more attractive approach is discussed in the following subsection.

### 2.2. Pixel-Level Techniques

**Figure 5.**
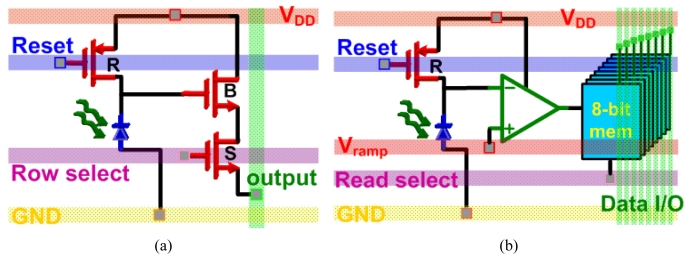
Simplified schematic diagrams of (a) a 3-T active pixel sensor (APS) and (b) a digital pixel sensor (DPS) containing part of the ADC and an 8-bit memory within the pixel.

Since digital transistors take more advantage of CMOS scaling properties, digital pixel sensors (DPS) have become very attractive. A DPS integrates an ADC into each pixel resulting in a massively parallel readout and conversion that can allow very high speed operation, where digital data is read out of each pixel. Figure 5 shows a simplified schematic representation of a standard 3-T active pixel sensor (APS) compared to a DPS. In this case, only part of the ADC is included within the pixel to maximize the FF, where an integrating ADC can be used with only one ramp generator and one counter that are common for all pixels. The in-pixel opamp compares the photodiode voltage to the ramp voltage (V_ramp_) and once V_ramp_ exceeds the photodiode voltage, the 8-bit memory cells will latch the count value that is coming in from the common counter. Using a DPS will only require one ADC conversion cycle for all pixels in parallel, which results in a great increase in FR, assuming that the readout circuits are fast enough to handle the extremely large amounts of data. The high speed readout makes CMOS image sensors suitable for very high-resolution imagers (multi-megapixels) especially for video applications.

The extra circuitry within the pixel in a DPS comes at the expense of reduced FF. However, the low FF of DPS sensors is no longer an issue for CMOS technologies of 0.18 μm and below [2, 15]. In 2001, Kleinfelder *et al.* [7] described a 352×288 pixel DPS imager in a 0.18 μm CMOS technology, with 37 transistors per pixel. The imager is capable of operating at 10,000 fps (1 Gpixel/s) with a power consumption of 50 mW and a pixel FF of 15%. Ghannoum *et al.* [8] improved the FF in 2007 to 26% by using a 90 nm CMOS technology with 57 transistors per pixel. The FR of a DPS-based imager can be calculated as:

(3) $${\text{FR}}_{\text{PP}-\text{ADC}}={\left[\tau_{\text{ADC}}+H\times V\times \frac{b}{n}\times \tau_{\text{RO}}\right]}^{-1}$$

**Figure 6.**
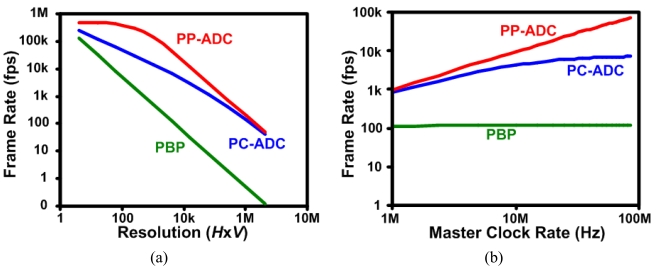
Simulation results of equations (1^3^) showing the FR of a pixel-by-pixel, PC-ADC and a PP-ADC readout architectures with 8-bit resolution ADCs (*b*=8), four 8-bit parallel outputs (*n*=32) and a *τ_ADC_* = 2 μ s. (a) The FR as a function of varying the imager resolution with a fixed clock rate of 50 MHz (1/*τ_RO_*). (b) The FR as a function of the clock rate with a fixed imager resolution of *H*×*V* = 64×64. Both graphs are shown in a log-log scale.

Unlike the pixel-by-pixel readout imager, the dominating factor that affects the FR of a DPS-based imager is the I/O transfer speed. Figure 6 shows a comparison between the frame rates of the pixel-by-pixel, PC-ADC and PP-ADC readout architectures based on equations (1^3^). From the figures, it can be seen that the FR of the pixel-by-pixel readout architecture is strongly affected by the resolution and there is an insignificant effect of the master clock rate. The PP-ADC and PC-ADC on the other hand are mainly affected by the readout speed and the advantage of using a DPS in PP-ADC readout as opposed to PC-ADC readout cannot be realized unless the chip I/O speed can handle the large data rates being generated. Figure 6 (a) shows that the PP-ADC has a constant FR versus resolution, until some point where the FR drops rapidly after the readout speed becomes too slow.

## 3. Analog Readout Architectures

Due to the bottleneck in chip I/O readout and ADC conversion times, even with PP-ADC, the published frame rates that use digital techniques are reaching their saturation limits. A number of researchers [10-14] explored analog readout methods for CMOS imagers. Stevanovic *et al.* and Hosticka *et al.* [10, 29] used imagers with 4 parallel analog output channels and 256×256 pixels achieving over 1,000 fps, while Lauxternann *et al.* [11] used 16 parallel analog output channels and 256×256 pixels to get a FR of 5,000 fps. Another approach to reduce the high-speed requirements of the ADC is to use an analog frame memory array [12]. By using an analog memory, the captured frame can be stored to separate the image capture and data conversion steps from each other. Sugiyama *et al.* [12] used this method for 3-D sensing and achieved a 320×240 pixels CMOS imager that can capture images at 3,300 fps.

**Figure 7.**
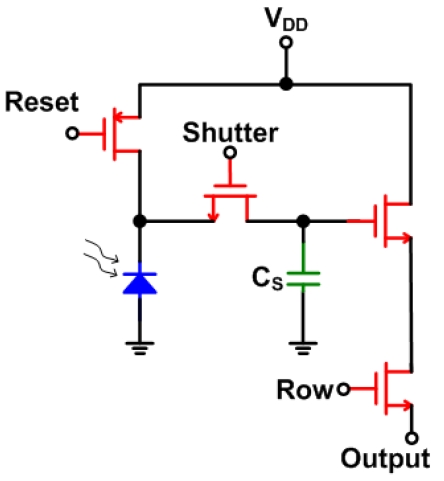
Simple 4-T APS with an analog storage element.

Analog memory techniques are even more interesting when the analog memory cell is included within the pixel. Including an analog memory unit within the pixel has been used in many imaging systems, with features such as motion detection [30], high dynamic range with pixel level integration time control [31], ambient light suppression [32] and cancellation of FPN or offset correction [33]. In-pixel memory is also used for high-speed applications to achieve imaging with a global shutter rather than a rolling shutter [5, 9, 10, 22 and 34]. Figure 7 shows a simple 4-T APS with a capacitor storage element (usually implemented using a MOS capacitor). Chapinal *et al.* [34] used the in-pixel storage capacitor to store the captured image for tens of seconds, avoiding the need for an external RAM. Dubois *et al.* [9] used two capacitors per pixel where one captures the current frame while the other holds the previous frame for processing, which increases the FR.

In order to achieve the fastest FR possible for a certain high-speed experiment, a number of extremely fast consecutive images can be captured and stored in analog form. By doing so, the inter-frame delay caused by the ADC conversion time and array readout can be avoided. In this case, the FR only depends on the speed of the devices and transistors used within the pixel, assuming a large enough illumination exists on the object being imaged. Depending on the type of experiment and the speed of capture, there will be a minimum number of frames that is acceptable.

**Figure 8.**
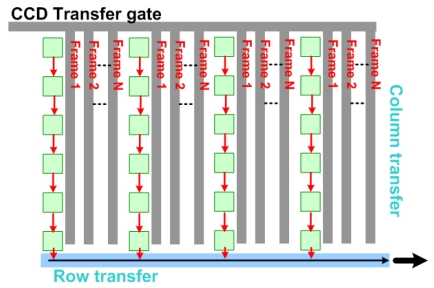
The storage and readout of an *in situ* CCD imager that can store up to N frames.

The concept of *in situ* storage has been implemented in both CCD imagers [13] and CMOS imagers [14]. Figure 8 shows the concept of an *in situ* CCD imager that stores up to N frames. By placing the storage elements in a very small area within or beside a pixel and increasing the number of storage elements as much as possible, which is equal to the number of consecutive frames, the theoretical maximum FR can be achieved [13]. The CCD *in situ* 312×260 pixel imager presented by Etoh *et al.* [13] can capture 100 consecutive images at a FR of 1M fps with a pixel FF of 13%.

A 1-D linear array CMOS implementation of the *in situ* imager has be presented by Kleinfelder *et al.* [14] in a CMOS 0.35 μm technology. The design has a 150 photodiodes with a 150-frame analog storage array and is capable of capturing images at a FR of 400 Mfps. The authors in [14] suggest using a 3-D packaging technique to achieve a 2-D imager design where chips are arranged standing on end with a separate photodiode array bonded on top.

## 4. Ultrahigh-Speed CMOS Imager

**Figure 9.**
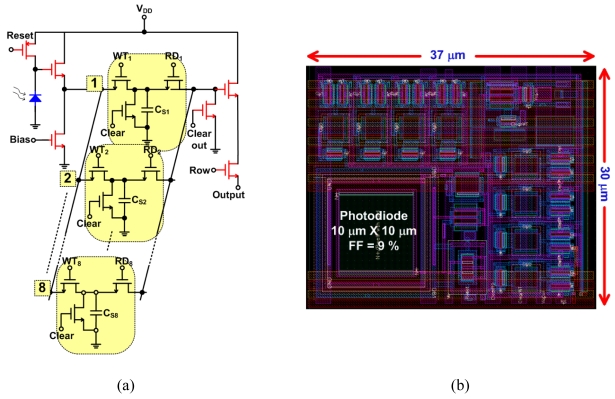
(a) The schematic diagram of the ultrahigh-speed *in-situ* APS containing 8 memory elements and 38 transistors and (b) the layout screen capture of a single pixel.

In this paper, we propose the design of an ultrahigh-speed APS that can capture 8 frames at an acquisition rate of 1.25 billion fps. The schematic diagram of the pixel, which contains 38 transistors, is shown in Figure 9 (a). The basic idea is to utilize 8 analog memory units *in situ* to temporarily hold 8 frames at a very high speed, avoiding the delay time in analog-to-digital conversion and readout. The write switches (WT) that select which storage element to use also serve as global shutters. The storage elements C_S1_ to C_S8_ were implemented using MOS capacitors to reduce layout area; they have a capacitance of 60 fF and were designed using thick-oxide devices to reduce leakage.

The pixel was designed using a CMOS 0.13 μm technology kit from IBM. Even though all devices were thick-oxide to increase the dynamic range, this kit can allow for a smaller pixel compared to the CMOS 0.18 μm technology. The imager contains a 32×32 pixel array. Figure 9 (b) shows a screen capture of the APS layout, which occupies an area of 37 μm × 30 μm. The photodiode used has an area of 10 μm × 10 μm, which gives a FF of 9%. The photodiode used is an n+/p_well with a guard ring, which was necessary to increase the speed of the photodiode by eliminating the slowly diffusing substrate carriers [35].

**Figure 10.**
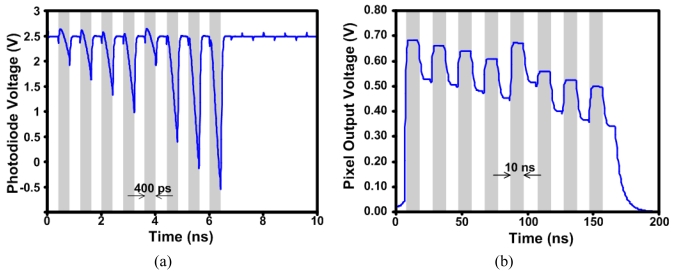
(a) Photodiode response for 8 different light samples. (b) Pixel readout voltage.

**Figure 11.**
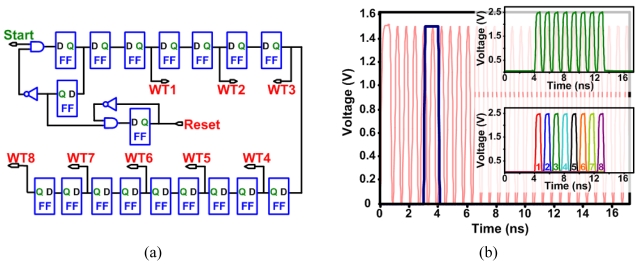
(a) Schematic diagram of the write and reset pulse generator circuit. (b) Simulation results of the write and reset pulse generator circuit showing the start pulse coming in at 3 ns with a clock frequency of 1.25 GHz. The top inset figure shows the 8 reset pulses (active low) and the bottom inset figure shows the generated 8 write pulse signals that have a width of 400 ps.

Figure 10 (a) shows a simulation test of the ultrahigh-speed APS, where the figure shows the photodiode voltage variation for 8 different light samples (simulated using a parallel ideal current source). Note that the 1^st^ and 5^th^ samples are the same and the reset frequency is 1.25 GHz. Figure 10 (b) shows the values read out of the pixel at a readout frequency of 50 MHz. An on-chip voltage-controlled oscillator (VCO) was designed to provide the high frequency write pulses and reset signals. The write pulses are generated by an edge-triggered circuit that accepts an external start pulse as an input (Figure 11 (a)).

Figure 11 (b) shows the simulation results of pulse generator circuit clocked at a frequency of 1.25 GHz. When the start pulse is received at 3 ns, the circuit generates the first write pulse (shown in the lower inset figure) and disables the reset transistor of the pixel. The circuit generates the 8 write pulses that have a width of 400 ps as well as the reset signal. The reset of the pixel is active low since a PMOS device was used.

## 5. Conclusions

CMOS technology has a great potential to be used in ultrahigh-speed imaging applications. Existing results with different CMOS imaging architectures have achieved thousands up to even millions of fps. Table 1 shows a summary of the different high-speed imager architectures discussed in this paper. By combining a number of different methods, which include parallel per-column or per-pixel ADCs, image compression, parallel output port readout, high readout clock rates and simultaneous capture and processing, researchers have managed to push frame-rates to 10,000 fps. Moving to higher frame-rates would require separating the acquisition and processing phases completely by relying on *in-situ* frame storage. Using an ultrahigh-speed imager design, such as the one discussed in Section 4, for a 1-D line-scan imager can increase the number of consecutive images that can be captured at rates of over a billion fps. A 2-D image can be coupled to the 1-D line-scan imager using fiber coupling to achieve ultrahigh-speed imaging without sacrificing the array fill-factor.

## Figures and Tables

**Table 1.** Summary of the various high-speed imagers available in the literature.

**REF.**	**TECH. (CMOS)**	**TECHNIQUE**	**PARALLEL OUTPUTS**	**CLOCK FREQ. (MHZ)**	**FR (fps)**	**ARRAY SIZE**	**PIXEL AREA**	**FF (%)**
[3]	0.5 μ m	PC-ADC & dual-portRAM	64	66	0.5k	1024×1024	(10 μ m)^2^	45
[4]	0.35 μ m	PC-ADC & dual-port RAM	160	66	0.24k	2352×1728	(7 μ m)^2^	43
[5]	0.25 μ m	PC-ADC & PC-compression	32	16.8	3k	256×256	(15 μ m)^2^	--
[6]	0.25 μ m	PC-ADC	160	68	3.5k	512×512	(20 μ m)^2^	--
[7]	0.18 μ m	PP-ADC	64	167	10k	352×288	(9.4 μ m)^2^	15
[8]	90 nm	PP-ADC	--	--	0.4k	64×48	(9 μ m)^2^	26
[9]	0.35 μ m	Smart-pixel	--	--	10k	64×64	(35 μ m)^2^	25
[10]	1.0 μ m	Parallel analog outputs	4	22	1.04k	256×256	(30 μ m)^2^	40
[11]	0.5 μ m	Parallel analog outputs	16	24	5k	256×256	9μ m×18μ m	42
[12]	0.35 μ m	Analog frame storage	--	--	3.3k	320×240	(11.2 μ m)^2^	53
[13]	CCD	*In-situ*	--	--	1M, 100-frames	312×260	(66.3 μ m)^2^	13
[14]	0.35 μ m	*In-situ*	--	--	10.5M, 64-frames	12×12	(200 μ m)^2^	--
This work	0.13 μ m	*In-situ*	8	50	1.25B, 8-frames	32×32	37μ m×30μ μμm	9

## References

[1] Charbon E. Will CMOS imagers ever need ultra-high speed?.

[2] Faramarzpour N., El-Desouki M.M., Deen M.J., Fang Q., Shrani S., Liu L.W.C. (2008). CMOS imaging for biomedical applications. IEEE Potentials.

[3] Krymski A., Van Blerkom D., Andersson A., Block N., Mansoorian B., Fossum E. R. (1999). A high speed, 500 frames/s, 1024 × 1024 CMOS active pixel sensor.

[4] Krymski A.I., Bock N.E., Tu N., Blerkom D.V., Fossum E.R. (2003). A high-speed, 240-frames/s, 4.1-Mpixel CMOS sensor. IEEE Trans. Electron Devices.

[5] Nishikawa Y., Kawahito S., Furuta M., Tamura T. (2007). A high-speed CMOS image sensor with on-chip parallel image compression circuits.

[6] Furuta M., Inoue T., Nishikawa Y., Kawahito S. (2006). A 3500fps high-apeed CMOS image sensor with 12b column-parallel cyclic A/D converters.

[7] Kleinfelder S., Lim S., Liu X., El Gamal A. (2001). A 10,000 frames/s CMOS digital pixel sensor. IEEE J. Solid-State Circuits.

[8] Ghannoum R., Sawan M. (2007). A 90nm CMOS multimode image sensor intended for a visual cortical stimulator.

[9] Dubois J., Ginhac D., Paindavoine M. (2006). A single-chip 10000 frames/s CMOS sensor with in-situ 2D programmable image processing.

[10] Stevanovic N., Hillegrand M., Hostica B.J., Teuner A. (2000). A CMOS image sensor for high-speed imaging.

[11] Lauxtermann S., Schwider P., Seitz P., Bloss H., Ernst J., Firla H. (1999). A high speed CMOS imager acquiring 5000 frames/sec.

[12] Sugiyama T., Yoshimura S., Suzuki R., Sumi H. (2002). A 1/4-inch QVGA color imaging and 3-D sensing CMOS sensor with analog frame memory.

[13] Etoh T.G., Poggemann D., Kreider G., Mutoh H., Theuwissen A.J.P., Ruckelshausen A., Kondo Y., Maruno H., Takubo K., Soya H., Takehara K., Okinaka T., Takano Y. (2003). An image sensor which captures 100 consecutive frames at 1000000 frames/s. IEEE Trans. Electron Devices.

[14] Kleinfelder S., Chen Y., Kwiatkowski K., Shah A. (2004). High-speed CMOS image sensor circuits with in-situ frame storage. IEEE Trans. Nucl. Sci..

[15] Campos F.S., Marinov O., Faramarzpour N., Saffih F., Deen M.J., Swart J.W. (2008). A multisampling time-domain CMOS imager with synchronous readout circuits. Analog Integr. Circuits Signal Process..

[16] Faramarzpour N., Deen M.J., Shirani S., Fang Q., Liu L. W-C., Campos F.S., Swart J.W. (2007). CMOS based active pixel for low-light-level detection: analysis and measurements. IEEE Trans. Electron Devices.

[17] Faramarzpour N., Deen M.J., Shirani S. (2006). An approach to improve the signal-to-noise ratio of active pixel sensor for low-light-level applications. IEEE Trans. Electron Devices.

[18] Kfouri M., Marinov O., Quevedo P., Faramarzpour N., Shirani S., Liu L. W-C., Fang Q., Deen M.J. (2008). Towards a miniaturized wireless fluorescence-based diagnostic imaging system. IEEE J. Sel. Top. Quantum Electron..

[19] Huang T., Sorgenfrei S., Shepard K.L., Gong P., Levicky R. (2007). A CMOS array sensor for sub-800-ps time-resolved fluorescence detection.

[20] Faramarzpour N., Deen M.J., Shirani S., Fang Q. (2008). Fully integrated single photon avalanche diode detector in standard CMOS 0.18μm technology. IEEE Trans. Electron Devices.

[21] Hogan G.E., Adams K.J., Alrick K.R., Amann J.F., Boissevain J.G., Crow M.L. (1999). Proton radiography.

[22] Bigas M., Cabruja E., Forest J., Salvi J. (2006). Review of CMOS image sensors. Microelectron. J..

[23] Kitchen A., Bermak A., Bouzerdoum A. (2005). A digital pixel sensor array with programmable dynamic range. IEEE Trans. Electron Devices.

[24] Bermak A. (2005). Conversion time analysis of time domain digital pixel sensor in uniform and non-uniform quantizers.

[25] Culurciello E., Etienne-Cummings R., Boahen K. A. (2003). A biomorphic digital image sensor. IEEE J. Solid-State Circuits.

[26] Culurciello E., Andreou A.G. (2006). CMOS image sensors for sensor networks. Proc. Analog Integ. Circuits.

[27] Culurciello E., Andreou A. G. (2004). ALOHA CMOS imager. IEEE Proc. Int. Symp. Cir. Sys. (ISCAS).

[28] Faramarzpour N., El-Desouki M.M., Deen M.J., Shrani S., Fang Q. (2007). CMOS photodetector systems for low-level light applications. J. Mater. Sci. - Mater. Electron..

[29] Hosticka B.J., Brockherde W., Bussmann A., Heimann T., Jeremias R., Kemna A., Nitta C., Schrey O. (2003). CMOS imaging for automotive application. IEEE Trans. Electron Devices.

[30] Simoni A., Torelli G., Maloberti F., Sartori A., Plevridis S., Birbas A. (1995). A single-chip optical sensor with analog memory for motion detection. IEEE J. Solid-State Circuits.

[31] Han S.-W., Kim S.-J., Choi J., Kim C.-K., Yoon E. (2006). A high dynamic range CMOS image sensor with in-pixel floating-node analog memory for pixel level integration time control. VLSI Circuits Symp. Dig..

[32] Ni Y., Yan X.L. (2002). CMOS active differential imaging device with single in-pixel analog memory.

[33] Aslam-Siddiqi A., Brockherde W., Schanz M., Hosticka B.J. (1998). A 128-pixel CMOS image sensor with integrated analog nonvolatile memory. IEEE J. Solid-State Circuits.

[34] Chapinal G., Bota S. (2002). A 128 × 128 CMOS image sensor with analog memory for synchronous image capture. IEEE Sens. J..

[35] Radovanovic S.A.J., Nauta B. (2005). A 3-Gb/s optical detector in standard CMOS for 850-nm optical communication. IEEE J. Solid-State Circuits.

